# Examining Parents’ Assessments of Objective and Subjective Social Status in Families of Children with Cancer

**DOI:** 10.1371/journal.pone.0089842

**Published:** 2014-03-05

**Authors:** Elizabeth A. Gage-Bouchard, Katie A. Devine

**Affiliations:** 1 Department of Community Health and Health Behavior, The School of Public Health and Health Professions, University at Buffalo, Buffalo, New York, United States of America; 2 Department of Medicine, Division of Population Sciences, Rutgers Cancer Institute of New Jersey, Rutgers, The State University of New Jersey, New Brunswick, New Jersey, United States of America; Newcastle University, United Kingdom

## Abstract

**Introduction:**

Understanding the social determinants of child health is a prominent area of research. This paper examines the measurement of socioeconomic position in a sample of families of children with cancer. Socioeconomic position is difficult to measure in pediatric health research due to sensitivity of asking about finances when research is conducted in health care delivery settings, financial volatility associated with periods of pediatric illness, and difficulty recruiting fathers to research.

**Methods:**

Caregivers of children with cancer (n = 76) completed a questionnaire that included the MacArthur Scale of Subjective Social Status (SSS). SSS was measured using two 10-rung ladders with differing referent groups: the US and respondents’ communities. Respondents placed themselves on each ladder by placing an X on the rung that represented their social position in relation to the two referent groups. Individuals’ SSS ratings and discrepancies in SSS ratings within couples were examined, and associations with objective social status measures were evaluated using Pearson correlations or t-tests.

**Results:**

Parents’ placement on the US and community ladders was positively associated with their income, education, wealth, household savings, and household savings minus debt. On average, respondents placed themselves higher on the US ladder compared to the community ladder. There was an average intra-couple discrepancy of 1.25 rungs in partner’s placements on the US ladder and a 1.56 rung difference for the community ladder. This intra-couple discrepancy was not associated with gender.

**Discussion:**

Results offer insight into the use of subjective social status measures to capture a more holistic assessment of socioeconomic position and the measurement of socioeconomic position in two-parent families.

## Introduction

Understanding the mechanisms that contribute to pediatric health disparities is a focus of research within medicine, public health, nursing and the social sciences, and scholars continue to examine the complicated relationships between family socioeconomic position, health behavior, health care experiences, and health outcomes in children [1]–[3]. Yet, there is not consensus about the best way to capture the multiple dimensions of socioeconomic position in research [4], [5]. Measuring socioeconomic position is particularly difficult in research on families because many studies only recruit one parent to participate [6], [7].

In this paper, we examine the measurement of socioeconomic position in a sample of families of children with cancer. A potentially life-threatening pediatric diagnosis represents one event over the life course that can contribute to difficulty in measuring socioeconomic position due to the financial and occupational volatility that may occur as parents take time out of the labor force or exhaust their resources to pay for expenses related to health care. These factors may further complicate the ability to make inferences about an families’ socioeconomic position based on traditional objective social status measures such as income, making alternative measures of social standing and socioeconomic status especially relevant for much pediatric health-related research.

Examining socioeconomic position in pediatric health-related research presents both theoretical and methodological challenges. The theoretical pathways through which a family’s socioeconomic position may influence health care behavior are multi-faceted and often difficult to capture empirically [4], [5]. For example, when coping with pediatric cancer, a family’s income may influence their ability to pay for health-related expenses, to take time out of the paid labor force, or to pay for help with household tasks to allow parents to focus their attention on their child’s cancer care. Parental education may influence knowledge of medical terms, ability to communicate with health care professionals, and understanding of treatment protocols. Finally, parents’ occupation may influence work schedule flexibility, access to paid time off, and fringe benefits [8]. Socioeconomic position may also shape the health care experience through the composition of families’ social networks, their social clout, and their social connections. Much health-related research uses income or education as a proxy for socioeconomic position; however, these variables may not capture the breadth of experiences, statuses, and skills inherent in socioeconomic position.

The use of objective measures of socioeconomic position such as income and education also presents methodological challenges. An individual’s income or level of education may be a sensitive issue [9], [10] and deemed inappropriate to ask about in certain research contexts. For example, when research is being conducted in a health care delivery setting, researchers may avoid asking patients directly about their income, finances, wealth, and debt due to concerns that patients may not distinguish the research from clinical assessment. Therefore, measures that capture socioeconomic position without asking about potentially sensitive topics, such as income, would allow more research conducted in clinical settings to capture data about respondents’ socioeconomic position and experiences.

Studying the experiences of families raises additional methodological questions about the appropriateness of inferring family socioeconomic position based upon the responses of one parent. It is often difficult to recruit fathers to participate in studies [6], [7], and much research relies on the responses of mothers. When studying two-parent families it is important to have a more complete understanding of how often parents’ reports of measures capturing socioeconomic position converge or diverge.

### Subjective Social Status

Given the limitations associated with measures of objective social status, measures of subjective social status (SSS) have been employed to capture a more complete representation of socioeconomic position. SSS measures capture individuals’ perceptions of their position within the social hierarchy and are thought to more holistically capture the nuances of social standing than traditional measures of objective social status (OSS) such as income and education [11]–[13]. SSS measures may capture a more comprehensive view of the resources families have available to cope with periods of serious pediatric illness. For example, a person’s perception of their status in his or her community may capture the social support available to help buffer stressful life events. Subjective social status is thought to represent individuals’ past experiences and expected future experiences, in addition to individuals’ current social circumstances [14]. SSS may also capture the psychosocial impact of social stratification, as well as the relative access to social resources that are captured in objective social status measures such as income and education [11]. For these reasons, scholars have suggested that SSS may be a better predictor of health behaviors and outcomes than traditional OSS measures [11]. SSS measures may capture aspects of social position that offer insight on the coping resources people have available above and beyond what is captured through traditional OSS measures such as income.

To capture subjective social status, researchers have traditionally asked respondents to identify their social class from a list provided, such as lower class, working class, middle class, upper middle class or upper class [15]. However, these measures require all respondents to have a similar conceptualization of social class categories [14], [16]. Recently, researchers have increasingly used ladders as an alternate way to capture self-identification in the social hierarchy [17]–[19]. The MacArthur Scale of Subjective Social Status asks respondents to place themselves on a “social ladder” by marking the rung on which they believe they stand [20].

The MacArthur Scale of Subjective Social Status includes two ladders, each with a different referent group to which respondents are asked to compare themselves [17]. The US ladder asks respondents to place themselves where they believe they stand in relation to the Unites States [20], and is thought to represent a traditional concept of social class position. Several studies have examined correlations between traditional objective measures of social status and respondents’ placement on the US ladder. These studies find that respondents’ placement on the US ladder is positively associated with their income, education and occupation [19], [21], [22]. Studies have also found an association between respondents’ US ladder position and health, with higher placement on the US ladder associated with better physical and mental health [14], [17], [19], [21], [24].

The second ladder in the MacArthur scale is the community ladder. In contrast to the US ladder, the community ladder employs a proximal referent group and asks respondents to place themselves where they stand in their communities [17]. The community ladder is designed to capture individuals’ more localized social positions. This distinction in referent groups is believed to have important theoretical implications, which may be especially relevant for poorer communities. For example, individuals may have limited income and educational attainment and therefore place themselves low on the US ladder. However, they may hold a prominent role in their church or neighborhood organizations, giving them high standing within their community [20]. Despite these important theoretical distinctions, few studies use both the US and community ladders [20]–[21]. In one study of middle-aged women, Ghaed and Gallo found that respondents’ US and community ladder rankings were significantly positively associated [21]. Similarly, in a study of adults in Taiwan, Goldman and colleagues found that respondents perceived themselves to have higher standing in their communities than in the national hierarchy [23]. On average, respondents placed themselves 0.4 rungs higher within their community than within the country of Taiwan [23].

Building on this research, we examine the measurement of socioeconomic position in a sample of parents of children with cancer. The first aim of this paper is to compare parents’ self-reports of social standing in the US and in their communities and examine what characteristics are associated with parents’ reporting of different social positions in the US and in their communities. We expect moderate to strong correlations between objective measures of socioeconomic status and the US ladder, and weaker correlations between objective measures of SES and the community ladder. Specifically, we examine these dynamics by analyzing the association between income, education, wealth, savings, debt, and parents’ responses to the US and community ladders.

While the MacArthur Subjective Social Status measures are becoming more widely used, studies have not yet compared how multiple respondents within the same household rate themselves on the US and community ladders. Yet, research on two-parent families commonly recruits one parent to participate in studies [6], [7]. In order to enhance understanding of the most appropriate measures to use when only one parent is recruited to a study, it is important to compare parents’ reports of both objective and subjective social status measures. The second aim of this paper is to compare partners’ responses to both objective and subjective social status measures. We use a sub-sample of cohabitating and married couples, in which both partners are included in the sample. We descriptively compare partners’ reports of objective socioeconomic status measures (such as household income and household wealth) as well as partners’ responses to the US and community ladders. We also explored the role of gender in parental differences.

## Methods

### Participant Recruitment

Participants were recruited through one NCI-designated comprehensive cancer center in the northeastern United States. To maintain patient privacy, the study team partnered with clinicians at the cancer center to facilitate participant recruitment. A pediatric psychologist or a social worker introduced the study to potential participants and asked parents to sign a form giving them permission to forward parents’ contact information to the study team. Eighty-two caregivers were approached and asked to participate. Seventy-six caregivers (93% of those approached) were enrolled in the study. The six not enrolled reported scheduling difficulties or declined to participate. Data were collected from August 2009 to May 2011. The study was approved by the Institutional Review Board at the recruitment site, and all participants provided written informed consent.

### Objective Socioeconomic Status Measures

To capture education, we asked respondents to indicate their highest degree earned. We utilized degree earned as an ordinal variable, ranking from less than high school to doctorate degree. Individual income was captured by asking respondents how much they earned before taxes and deductions in the previous 12 months. Response options ranged from less than $5,000 to $100,000 and greater. Total household income was measured by asking respondents how much they and members of their household earned form all sources in the previous 12 months. Response options ranged from less than $5,000 to $100,000 and greater. We used three measures from the MacArthur Foundation Research Network on Socioeconomic Status and Health socio-demographic questionnaire to capture different dimensions of household assets [20]. Wealth was captured by asking respondents, “If you lost all your current source(s) of household income (your paycheck, public assistance, or other forms of income), how long could you continue to live at your current address and standard of living?” Response options included less than one month, 1–2 months, 3–6 months, 7–12 months, and more than 1 year. To assess total household savings we asked respondents, “Suppose you needed money quickly and you cashed in all of your (and your spouse’s) checking and savings accounts, and any stocks and bonds. If you added up what you would get, about how much would this amount to?” Response options ranged from less than $500 to $500,000 and greater. Finally, to capture household savings minus debt we asked, “If you now subtracted out any debt that you have (credit card debt, unpaid loans including car loans, home mortgage), about how much would you have left?” Response options ranged from less than $500 to $500,000 and greater.

### Subjective Social Status Measures

To collect data on subjective social status, we used measures from the MacArthur Foundation Research Network on Socioeconomic Status and Health socio-demographic questionnaire [20]. Subjective social status was measured using two 10-rung ladders. The US ladder asked people to place themselves on a 10-rung ladder representing the United States and provided the following instructions:

Think of this ladder as representing where people stand in the United States. At the top of the ladder are the people who are the best off – those who have the most money, the most education, and the most respected jobs. At the bottom are the people who are the worst off – who have the least money, least education, and the least respected jobs or no job. The higher you are on this ladder, the closer you are to the people at the very top; the lower you are, the closer you are to the people at the very bottom. Where would you place yourself on this ladder?

The community ladder gave the following instructions:

Think of this ladder as representing where people stand in their communities. People define community in different ways; please define it in whatever way is most meaningful to you. At the top of the ladder are the people who have the highest standing in their community. At the bottom are the people who have the lowest standing in their community. Where would you place yourself on this ladder?

Respondents placed themselves on each ladder by placing an X on the rung that represented their social position in relation to the two referent groups.

### Procedures

The study was IRB approved, and all respondents provided written informed consent. Study team members met respondents at locations of their choice, including the hospital if their child was admitted, respondents’ homes, and coffee shops, to administer the survey. Participants were compensated with a $50 gift card after completing the survey. In the case of families in which both partners participated in the study, individual appointments were scheduled with each respondent. All respondents completed the surveys separately, without their partner in the room. To protect respondent confidentiality, no further data are publically available.

### Participants

Seventy-six caregivers (45 mothers, 28 fathers, 3 aunts) of pediatric cancer patients participated in the study. Of the 76 caregivers, 52 (68%) were from two-parent families. Parent characteristics for the full sample and for the subsample of two-parent families are presented in Table 1. For the full sample, 64% of respondents were women, and 79% were non-Hispanic white. Seventy percent of respondents in the full sample were married, and 54% had less than a bachelor’s degree. There were some expected differences in demographic trends between the sub-sample of two-parent families and non-two-parent families. Men comprised a higher percentage of respondents in the sub-sample of two-parent families compared with non-two parent families, χ^2^(1) = 15.06, *p*<.01. The sub-sample of two-parent families were more likely to have earned a bachelor’s degree or more, χ^2^(1)  = 6.43, *p*<.05, reported higher income, *t*(70)  = 2.39, *p*<.05, and reported higher household savings, *t*(62)  = 2.04, *p*<.05, compared with non-two-parent families.

**Table 1:** Demographic Characteristics of Sample.

	Total Sample	Two-Parent Families
	*N* = 76	*n* = 52
	% (n)	% (n)
**Caregiver Characteristics**		
Gender**		
Women	64% (49)	50% (26)
Men	36% (27)	50% (26)
Total	100% (76)	100% (52)
Race/Ethnicity		
Non-Hispanic White	79% (60)	83% (43)
Non-Hispanic Black	21% (16)	17% (9)
Total	100% (76)	100% (52)
Marital Status^a^**		
Never married	18% (14)	12% (6)
Married	70% (53)	81% (42)
Divorced/Separated	12% (9)	8% (4)
Total	100% (76)	100% (52)
Education^b^*		
Less than high school diploma	2% (2)	4% (2)
High school diploma or equivalency (GED)	41% (31)	33% (17)
Associate degree (junior college)	11% (8)	6% (3)
Bachelor's degree	22% (17)	33% (17)
Master's degree	19% (14)	19% (10)
Doctorate or advanced professional degree	1% (1)	0% (0)
No Response	4% (3)	6% (3)
Total	100% (76)	100% (52)
Total Household Income*		
Less than $5,000	5% (4)	2% (1)
$5,000 through $11,999	8% (6)	8% (4)
$12,000 through $15,999	4% (3)	4% (2)
$16,000 through $24,999	9% (7)	6% (3)
$25,000 through $34,999	3% (2)	0% (0)
$35,000 through $49,999	12% (9)	15% (8)
$50,000 through $74,999	13% (10)	15% (8)
$75,000 through $99,999	12% (9)	6% (3)
$100,000 and greater	29% (22)	39% (20)
Don’t Know/No Response	5% (4)	6% (3)
Total	100% (76)	100% (52)
**Wealth**		
Less than 1 month	24% (18)	19% (10)
1–2 months	16% (12)	21% (11)
3–6 months	30% (23)	29% (15)
7–12 months	12% (9)	15% (8)
More than 1 year	17% (13)	15% (8)
Don’t Know/No Response	1% (1)	0% (0)
Total	100% (76)	100% (52)
Household Savings*		
Less than $500	21% (16)	15% (8)
$500 to $5,000	16% (12)	15% (8)
$5,000 to $9,999	4% (3)	4% (2)
$10,000 to $19,999	7% (5)	4% (2)
$20,000 to $49,999	12% (9)	15% (8)
$50,000 to $99,999	8% (6)	12% (6)
$100,000 to $199,999	9% (7)	12% (6)
$200,000 to $499,999	7% (5)	6% (3)
More than $500,000	1% (1)	2% (1)
Don’t Know/No Response	15% (12)	4% (2)
Total	100% (76)	100% (52)
**Household Savings Minus Debt**		
Less than $500	51% (39)	44% (23)
$500 to $5,000	3% (2)	2% (1)
$5,000 to $9,999	3% (2)	4% (2)
$10,000 to $19,999	4% (3)	6% (3)
$20,000 to $49,999	4% (3)	6% (3)
$50,000 to $99,999	5% (4)	6% (3)
$100,000 to $199,999	4% (3)	6% (3)
$200,000 to $499,999	4% (3)	2% (1)
Don’t Know/No Response	22% (17)	25% (13)
Total	100% (76)	100% (52)
** US Ladder Rung^c^**		
1 & 2	2% (2)	2% (1)
3 & 4	27% (20)	28% (15)
5 & 6	37% (28)	44% (23)
7 & 8	18% (13)	12% (6)
9 & 10	12% (9)	10% (5)
No Response	4% (3)	4% (2)
Total	100% (76)	100% (52)
**Community Ladder Rung^c^**		
1 & 2	4% (3)	2% (1)
3 & 4	34% (26)	40% (21)
5 & 6	34% (26)	34% (18)
7 & 8	21% (16)	18% (9)
9 & 10	4% (3)	4% (2)
No Response	3% (2)	2% (1)
Total	100% (76)	100% (52)

*Note.* Differences in demographics between subsample of two-parent families and non-two-parent families are noted, *p<.05, **p<.01. ^a^Marital status was dichotomized as “married” vs. “not married” for comparison of subsamples; ^b^Educational attainment was dichotomized as “less than bachelor’s degree” vs. “bachelor’s degree or greater” for comparison of subsamples; **^c^**1&2 are high subjective social status categories, 9&10 are low.

Table 2 presents child characteristics for the full sample and the sub-sample of two-parent families. Forty six percent of parents in the full sample were caring for a male child diagnosed with cancer, and 36% of respondents were caring for a child diagnosed with a solid tumor cancer. The mean time since child diagnosis for the full sample was 2.3 years. In the geographic area where the study took place, children who were previously uninsured were eligible for Social Security Insurance (SSI) and Medicaid due to their cancer diagnosis. Therefore, all children in the sample had either private health insurance or Medicaid.

**Table 2:** Child Characteristics.

	Total Sample	Two-Parent Families
	*N* = 76	*n* = 52
	% (n)	% (n)
**Gender**		
Male	46% (35)	42% (22)
Female	54% (41)	58% (30)
Total	100% (76)	100% (52)
**Diagnosis**		
Solid Tumor	36% (27)	35% (18)
Cancer of the Blood	59% (45)	62% (32)
Don’t Know/No Response	5% (4)	4% (2)
Total	100% (76)	100% (52)
**Age at Diagnosis (*M* ± *SD*)**	7.9±4.7 yrs	8.1±4.5 yrs
**Current Age (*M* ± *SD*)**	10.2±4.8 yrs	10.5±4.9 yrs
**Time Since Diagnosis (*M* ± *SD*)**	2.3±4.3 yrs	2.4±4.2 yrs

### Statistical Analysis

Preliminary analyses were conducted to analyze the distributions of the data and check for data entry errors. Visual inspection of histograms and normal Q-Q plots indicated that responses on the US and community ladders were approximately normally distributed, with a skewness of –0.24 (SE = .28) and kurtosis of –0.42 (SE = .56) for the US ladder and a skewness of –0.14 (SE = .28) and kurtosis of –0.33 (SE = .56) for the community ladder.

For the first aim, we examined individuals’ responses to the US and community ladders via Pearson correlations with quantitative variables (income, degree earned, wealth, household savings, and household savings minus debt). We examined any differences by race or gender using independent samples t-tests. If race was significantly associated with an outcome, we conducted an ANCOVA controlling for measures of objective social status (income and education). We calculated the difference between individuals’ responses on the US and community ladders by subtracting the rung reported for the US ladder from the rung reported for the community ladder (thus, positive differences would indicate a higher placement within the community compared to the US). We evaluated whether individuals on average reported a discrepancy between ladders using a one-sample t-test with a test value of 0, which would indicate no difference between ladders. We then examined factors associated with differences using Pearson correlations for quantitative variables and independent samples t-tests for race and gender. We compared the magnitude of association between the measures of objective social status and the US ladder vs. the Community ladder by testing the difference between the two correlation coefficients. Steiger’s Z method (Steiger 1980) was used, which compares two correlation coefficients that share a common variable (i.e., the objective measure of social status). Steiger’s Z and the 95% CI around the difference were calculated using SISA (Uitenbroek, 1997).

For the second aim, family income, wealth, household savings, and household savings minus debt were the outcomes for the objective measure of social status, and responses on the US and community ladders were the outcomes for subjective measures of social status. We descriptively examined intra-couple differences by taking the absolute value of the number of categories between parents’ reports of income, wealth, household savings, household savings minus debt, US ladder standing, and community ladder standing. To examine the possible role of gender in reporting of SSS, we calculated the difference in reports of the male from the female in the relationship and compared the average gender difference to zero using a one-sample t-test.

## Results

### Aim 1: Compare individuals’ self-reports of social standing in the US and in their communities and examine what characteristics are associated with individuals’ reporting of different social positions in the US and in their communities

First, we examined the Pearson correlations between individuals’ responses on the US and community ladders. There was a significant, positive association between responses to the US and community ladders, *r* = .58, *p*<.001. Responses on the US and community ladders were significantly and positively associated with measures of objective social status (income, degree earned, wealth, household savings, and household savings minus debt; see Table 3). The magnitude of association with the US ladder was significantly greater than the association with the community ladder for income (*Z* = 2.14, *p* = .03), wealth (*Z* = 2.34, *p* = .02), household savings (*Z* = 3.06, *p* = .002), and household savings minus debt (*Z* = 2.35, *p* = .02), but not for education (*Z* = 1.73, *p* = .08). There were differences in reporting on the US ladder according to race, with non-Hispanic White participants on average reporting 1.71 rungs higher than non-Hispanic Black participants, *t*(71)  = 3.14, *p* = .002, 95% CI [0.62, 2.80]. However, when income and degree earned were controlled for, race was no longer a significant predictor of US ladder response, *F*(1, 62)  = 0.30, *p* = .59. There were no significant differences by race on the community ladder, and no significant differences by gender.

**Table 3:** Pearson correlations or t-tests between objective measures of social status or demographic factors and subjective measures (US and Community Ladders).

Objective Measure/Demographic	US Ladder	Community Ladder	Difference
			[95% CI]^a^
Income	.65**	.46**	0.19 [0.02, 0.36]*
Degree Earned	.53**	.36**	0.17 [–0.03, 0.37]
Race	*t*(71) = 3.14**	*t*(72) = 1.92	---
Gender	*t*(71) = 0.72	*t*(72) = 0.91	---
Wealth	.58**	0.36**	0.22 [0.03, 0.41]*
Household Savings	.66**	.36**	0.30 [0.10, 0.50]**
Household Savings Minus Debt	.54**	.28*	0.26 [0.04, 0.48]*

*Note.* **^a^**Steiger’s Z was used to evaluate the significance of the difference between two correlation coefficients that share a common variable.

*p<.05, **p<.01.

On average, participants rated their community standing as 0.59 rungs above their US standing, *t*(72)  = 2.75, *p* = .008, 95% CI [0.16, 1.02]. Differences between individuals’ responses on the community and US ladders were significantly inversely associated with wealth, *r* = –.26, *p* = .03, household savings, *r* = –.33, *p* = .01, and household savings minus debt, *r* = –.27, *p* = .04, indicating that a larger difference between the ladders was associated with lower levels of objective measures of wealth and savings. Differences between individuals’ responses on the community and US ladders were not significantly associated with income, degree earned, race, or gender.

### Aim 2: Compare partners’ responses to both objective and subjective social status measures

With regard to the objective measure of income, the majority of parents in two-parent families reported the same income category (57.7%; see Table 4). On average, parents reported a 0.48 difference in income categories. For wealth, couples on average reported a 1.12 category discrepancy. Half of the couples reported a one category difference (see Table 4). With regard to household savings, parents in two-parent families reported an average of a 0.79 category difference, with a 0 category difference being the most common response (38.5%). For household savings minus debt, the average category discrepancy between parents in two-parent families was 1.40. It is notable that there was a large amount of missing data from at least one respondent for household savings (26.9%) and household savings minus debt (42.3%).

**Table 4:** Within-couple differences in reporting of objective and subjective measures of social status.

Category Difference	Income	Wealth	Household Savings	Savings Minus Debt	US Ladder	Community Ladder
0	57.7%	19.2%	38.5%	26.9%	19.2%	15.4%
1	19.2%	50.0%	19.2%	7.7%	42.3%	38.5%
2	11.5%	30.8%	7.7%	7.7%	19.2%	19.2%
3	--	--	7.7%	7.7%	11.5%	19.2%
4	--	--	--	3.8%	--	3.8%
5	--	--	--	3.8%	--	--
Missing	11.5%	--	26.9%	42.3%	7.7%	3.8%

*Note.* N = 26 couples.

In terms of subjective social status measured by the US ladder, a one-rung difference on the US ladder was most common (42.3%; see Table 4). On average, parents reported a 1.25 difference in rungs. There was not a statistically significant difference by gender, *t*(23)  = –0.26, *p* >.05. On the community ladder, a one-rung difference again was most common (38.5%; see Table 4). On average, parents reported a 1.56 difference in rungs. There was not a statistically significant difference by gender, *t*(24)  = –0.31, *p* >.05.

## Discussion

In this study, we examined the measurement of socioeconomic position in a sample of families of children with cancer. Measuring socioeconomic position in research in which respondents are experiencing an acute pediatric health crisis presents practical and methodological dilemmas. Clinicians are often reluctant to include items assessing families’ income and finances due to their potential sensitivity in research conducted in patient care settings. Periods of acute pediatric illness can also be related to financial volatility and child illness may influence parents’ work force participation, as well as the proportion of income and savings spent on medical expenses. For these reasons, measures of subjective social status may offer a preferable alternative and add valuable information regarding socioeconomic position for families experiencing a serious pediatric illness beyond traditional measures of objective social status. Our first aim in this paper was to compare how respondents rated their SSS when provided with two referent groups – the US and their community – and to examine which indicators of objective social status were associated with parents’ self-reported SSS. In our sample, respondents’ placement on both the US ladder and community ladder were positively associated with their income, degree earned, wealth, household savings, and household savings minus debt, but all OSS measures except degree earned were more strongly related to the US ladder than the Community ladder. This finding is consistent with previous research showing objective measures of social status to be more strongly related to the US ladder [22]. It is also consistent with the theoretical basis for providing two referent groups and shows face validity of the ladders, as respondents appear to be taking objective measures of social status into consideration when rating their US standing. Other studies have also found positive associations between ratings on the US ladder and education, occupation, and annual income [21]. In this previous research, however, women’s perception of their standing in their communities was not significantly associated with their education, contrary to our findings [21].

We were specifically interested in understanding the context in which parents report different SSS on the US ladder compared to the community ladder. We found that on average, respondents placed themselves 0.59 rungs higher on the community ladder than on the US ladder. This finding is consistent with other studies comparing respondents’ perceptions of community and US SSS [11]–[12]. We found that differences in individuals’ placement on the US and community ladders was inversely associated with their wealth, household savings, and household savings minus debt. Therefore, respondents were more likely to report higher standing in their communities versus the United States if they had lower levels of access to non-income financial resources. Scholars have speculated that the distinction between the US and community referent groups may lead to the capture of different nuances of social standing, with the US ladder measuring a more traditional SES construct and the community ladder measuring respondents’ self-esteem or perceived social support [11], [21]. Ghaed and Gallo suggest that community SSS may more accurately assess “…an individual’s comprehensive understanding of his or her standing within a context of past, present, and future accomplishments” [21: 673]. It may be that one of the important distinctions in how respondents’ rate their social standing in the US compared to their community may be related to their wealth, savings, and debt. These variables may capture individuals’ past, present, and future social positions, rather than income, which may fluctuate over time.

To our knowledge, studies have not yet examined how multiple members of the same household rate their subjective social status. Yet, it is important to understand how often partners’ assessments of SSS converge or diverge since many studies on two-parent families recruit only one parent to participate in the research. We first examined if parents in two-parent families reported different objective social status, measured by total household income, wealth, household savings, and savings minus debt. In our sample, most respondents in two-parent families (57%) reported the same total household income. However, small but sizeable percentages of couples reported relatively large differences in OSS. That is, 11.5% of respondents reported a two category difference in their total household income, 30.8% of respondents reported a two category difference in their wealth, and 23% reported a two to five category difference in savings minus debt. Differences in parental reports of objective social status may be due to the fluctuation of objective social status markers during medical treatment for pediatric cancer. All of the respondents in the sample had a child who had been diagnosed with pediatric cancer. Thus, their income, savings, and debt may have been changing at the time of data collection because parents may have taken time off from the paid labor force or used savings to pay for their child’s care. These issues of volatility in measures of objective social status during periods of severe illness highlight the potential utility of subjective social status measures in pediatric health-related research. In terms of assessing household savings and household savings minus debt, there were large amounts of missing data due to one or both parents not providing a response. This could indicate a division of responsibility for savings management between parents or a difficulty in assessing current savings and accounting for current debts. Alternatively, it could suggest that these more specific measures of objective social status are difficult for participants to answer.

In terms of discrepancies in intra-couple ratings on the US ladder, there was a 1.25 rung average difference in parents’ placement on the ladder. For the community ladder, there was a 1.56 rung average difference in parents’ placement on the ladder. The most common difference reported was 1 rung for both ladders. Gender did not seem to influence reporting. This finding that parents in two-parent families reported intra-couple differences in their standing in both the US and communities may lend support to the hypothesis that subjective social status is a more complete representation of individuals’ social status throughout their life course. Unlike total household income, which captures a family’s current financial means, the SSS ladder ranking may reflect respondents’ past experiences and their expectations for the future. For example, when responding to SSS measures, individuals may recall their family environment in childhood or the standing of the family they have married into [22]. This may account for partners’ differential assessments of their standing in the US and in their communities. Alternatively, one partner may have a higher status occupation or higher level of education, and these differences may account for their divergence in SSS ratings.

Both the US and community ladders use a 10-point response scale which may capture greater nuance in individual’s social position than traditional measures of objective social status [22]. For example, differences in individuals’ placement on the ladders may capture the importance of not just obtaining a college degree, but the prestige of the institution where that degree was obtained [22]. Alternatively the ladders may capture more subtle differences in occupational prestige between occupations that are commonly categorized together in traditional measures of SES, such as physician assistant and physician. The average intra-couple differences of 1.25 on the US ladder and 1.56 on the community ladder may capture these nuances of social standing within families.

The ladders ask respondents to place themselves, and not their families, where they believe they stand in relationship to the US and their communities. Differences in income, occupation, or education between spouses or partners may shape different perceptions of individual standing in the US and their communities. Due to this, these measures of subjective status may offer valuable information regarding the social resources individual family members possess, above and beyond traditional objective measures such as total household income.

### Limitations

This study contributes to the interpretation of subjective social status measures that are increasingly common in health-related research. However, there are some limitations to note when interpreting findings. First, this study did not measure health outcomes so we were unable to examine the influence of OSS and SSS measures on healthcare utilization or measures of child or parent well-being. All respondents live in the same northeastern city and had a child diagnosed with pediatric cancer. While the sample had a wide distribution of respondents across the SES spectrum, the findings may not be generalizable to individuals living in other areas of the United States or to parents of healthy children. Nevertheless, we found similar patterns as previous research, suggesting that these findings may not be idiosyncratic to this study population [11]–[12], [21]. Future research should also examine whether these results would generalize to families facing similar financial volatility due to caring for a seriously ill family member, such as families of children with other serious illnesses or families of elderly patients with health problems. While the comparison of intra-couple reports of SSS refines our understanding of the SSS construct, our sub-sample of two-parent families was small, limiting our ability to evaluate predictors of intra-couple differences. Additionally, we had a small proportion of non-white families in our sample. Therefore, future studies should examine intra-couple reports of OSS and SSS in larger samples with more racial and ethnic diversity.

We did not find intra-couple gender differences in SSS reports. Our sample was comprised of parents with younger families, and respondents in our sample may have more egalitarian conceptions of gender. Another possibility is that this may be an effect of our small sample size. The sample may have been underpowered to detect small effects.

Our results indicating differences within partners’ reports of SSS provide a starting point for future research. While we suspect that intra-couple divergence in SSS reports may be due in part to experiences throughout the life course, we did not include measures of past OSS or SSS in this study and therefore could not explicitly examine this hypothesis. Future research should include measures of past OSS and SSS to refine our understanding of these processes. It would also be particularly interesting to follow families from diagnosis over time to determine whether changes in OSS or SSS occur and try to identify whether such changes are related to any medical factors, including the course of cancer treatment and survivorship. Future research using a mixed-methods design to include qualitative interviews with respondents about how they assess their SSS and how they interpret referent groups would substantially enhance our understanding of how to interpret SSS measures. Qualitative interviews would also permit examination of meaningful cut points within the SSS ladders and enhance interpretation of the relative difference in social standing represented by rungs on the ladders. Finally, qualitative interviews would permit examination of respondents’ perceptions of the invasiveness of questions asking them to reflect on their standing in the US and their communities. For our study, surveys were administered in-person, and no respondents voiced concerns or hesitations about completing the ladder items to study team members while completing the survey. However, directly asking respondents how they perceive the invasiveness of these ladder questions compared to more commonly used income or wealth items would add valuable insight on the appropriates of using the SSS ladder measures in various research contexts.

## Conclusions

To our knowledge, this is the first study to examine SSS measures in a sample of families coping with a serious pediatric illness. Results offer insight into the use of subjective social status measures to capture a more holistic assessment of socioeconomic position in research on the social determinants of child health. We found that respondents’ placement on both the US ladder and community ladder were positively associated with their income, degree earned, wealth, household savings, and household savings minus debt, though the strength of association was significantly greater for the US ladder for all variable except degree earned. Respondents who had lower wealth, household savings, and household savings minus debt were more likely to report differences in their placement on the US and community ladders. This finding has important implications for the design of future studies and for understanding the resources people may have available to buffer periods of poor health. The community ladder may be especially salient for individuals who have lower socioeconomic position when comparing themselves to the broad US population, yet they have higher standing in their communities that may offer resources that help them cope with periods of poor health or other life events. A parent’s perceived standing in their community may be reflective of their access to material, logistic, and emotional support. Therefore, for families coping with a serious pediatric illness, such as pediatric cancer, the community ladder may be an especially important tool to capture distinct dimensions of social and economic resources compared to those captured through measures of income, education, and the US ladder.

These results also offer insight into measuring family socioeconomic position in studies that rely on only one parent’s participation. Gender was not a significant predictor of intra-couple differences in SSS rating, suggesting that there were not systematic differences between mothers and fathers. When comparing partners’ reports, the highest percentage of couples reported a zero or one category difference, but sizeable percentages reported two or more category differences in total household income, household wealth, household savings, and household savings minus debt. In terms of discrepancies in intra-couple ratings on the US ladder, there was a 1.25 rung average difference in parents’ placement on the ladder, and a 1.56 rung average difference in parents’ placement on the community ladder. These differences suggest that it may not be appropriate to infer household socioeconomic position based upon one parent’s SSS rankings. Future studies seeking to capture household SSS may benefit from a using a ladder which asks respondents to rank their family, rather than individual, social standing.
